# Evolution and Functional Dynamics of the BAG Gene Family in Passion Fruit (*Passiflora edulis*)

**DOI:** 10.3390/plants14182887

**Published:** 2025-09-17

**Authors:** Munsif Ali Shad, Songguo Wu, Yuxin Wu, Lijie Zhang, Yuhong Zhou, Jingzheng Wang, Lingqiang Wang, Chongjian Ma, Lihua Hu

**Affiliations:** 1 Guangdong Provincial Key Laboratory of Utilization and Conservation of Food and Medicinal Resources in Northern Region, Shaoguan 512005, China; p2022027@gxu.edu.cn (M.A.S.); zhou_yuhong@sgu.edu.cn (Y.Z.);; 2 State Key Laboratory for Conservation and Utilization of Subtropical Agro-Bioresources, Guangxi University, Nanning 530004, Chinazhanglijie4421@163.com (L.Z.); 3 College of Life Sciences and Technology, Huazhong University of Sciences and Technology, Wuhan 430074, China; u202213718@hust.edu.cn; 4 College of Plant Science and Technology, Huazhong Agricultural University, Wuhan 430070, China; 5 Centre for Crop Science, Queensland Alliance for Agriculture and Food Innovation, The University of Queensland, St Lucia, Brisbane 4072, Australia

**Keywords:** passion fruit, BAG family, abiotic stress, subcellular localizations, development

## Abstract

B-cell lymphoma 2 (Bcl-2)-associated athanogene (BAG) family proteins are co-chaperones that regulate growth, development, and cell death and are evolutionarily conserved across eukaryotes. The BAG gene family has attracted intense interest in human health research, but its plant counterparts have received little attention. In this study, we conducted a genome-wide scan of the BAG family in passion fruit, a crop of great economic importance. Fifteen *PeBAG* genes were identified, with all but *PeBAG5* having multiple copies. PeBAG members, each characterized by a BAG domain, were categorized into two groups: Group 1 (PeBAG1/2/3/4) with extra ubiquitin domains, and Group 2 (PeBAG5/6-1/6-2) with additional plant-specific calmodulin-binding domains. The transcriptome data suggest that *PeBAGs* exhibit high gene expression in stems, flowers, and fruit, while *PeBAG4-3* and *6-1* upregulated under hormonal and abiotic stresses. qRT-PCR further confirmed the heat stress activation of *PeBAG4-3* and *6-1*. Subcellular localization in planta revealed varied compartmentalization, with members in the nucleus, cytoplasm, and plastids demonstrating functional divergence. This study provides a guide for investigating and employing *PeBAG* genes to improve heat resistance in passion fruit.

## 1. Introduction

Passion fruit (*Passiflora edulis* Sims/f. flavicarpa) is a tropical and scarce fruit vine belonging to the *Passiflora* genus of the Passifloraceae family. Its fruit pulp has about 100 different types of aroma. The vine is perennial and evergreen. It is cultivated worldwide, particularly in the United States, Australia, and Africa, and is native to central and northern South America [1]. Due to its unique flavor and brief growth cycle, China and its neighboring countries are experiencing significant growth in cultivated areas [2]. In addition to being nutritious, passion fruit has significant medical benefits. The entire plant possesses antibacterial, anti-inflammatory, antioxidant, and antifungal properties, which help treat conditions such as diabetes and pulmonary fibrosis, as well as control and prevent obesity and its associated medical issues [3,4,5]. The challenges of cultivating passion fruit are increasing alongside its yield expansion. Abiotic stressors seriously threaten the growth of the passion fruit industry. Therefore, it is essential to explore the traits of stress-resistant genes in passion fruit and understand their mechanisms of action [6,7,8].

The Bcl-2-associated athanogene (BAG) is a group of evolutionarily conserved proteins that function as co-chaperones in plants [9] and animals [10], playing a role in various cellular activities and diverse physiological processes. BAG proteins share a common conserved region near the C-terminal known as the BAG domain (BD), which binds to the ATPase domain of Hsp70/Hsc70 molecular chaperones [11]. The N-terminal, on the other hand, interacts with multiple molecular chaperones, forming a ternary complex and serving as a “bridge” for molecular chaperones [12]. Human skeletal muscle cells and cardiomyocytes constitutively express BAG3, which helps cells withstand mechanical stress [13]. BAG proteins are involved in various cell functions and physiological processes, including division, migration, apoptosis, autoimmune responses, tumorigenesis, neuronal differentiation, stress responses, and the cell cycle in animals [14,15].

Similarly, BAG genes are vital constituents of the broader stress response pathways in plants. For instance, *AtBAG6* and *AtBAG2* act as positive regulators of heat stress, whereas they play a negative role in drought stress [16]. BAG proteins facilitate protein folding and protect against stress-induced damage through their interactions with heat shock proteins (HSPs) [17]. T-DNA insertion mutant lines for *AtBAG6* showed a lack of basal thermotolerance, whereas transgenic GUS lines expressing *AtBAG6* promoters exhibited increased activity at elevated temperatures [18]. On the contrary, *osbag6* knockout mutants showed reduced sensitivity to alkaline-saline stress. In contrast, transgenic overexpression lines harboring *OsBAG6* exhibited increased sensitivity to alkaline-saline stress, suggesting it acts as a negative regulator for alkaline-saline stress endurance in rice [19]. Similarly, ectopic overexpression of tomato *SlBAG9* enhanced the sensitivity of transgenic *Arabidopsis* seedlings to salt, ABA, and drought during germination and seedling growth [20]. In rice, *OsBAG4* positively affected salt stress through its action as a link between OsMYB106 and OsSUVH7, inducing the attachment of the former protein to its binding cis-regulatory element in the OsHKT1;5 promoter and thereby inducing its transcription [21].

Closely aligned to their roles in stress response, BAG genes are crucial for plant growth and development. They are involved in maintaining cellular homeostasis and regulating programmed cell death (PCD) during developmental stages [22]. BAGs are also engaged in organelle degradation. *SlBAG5b* and *SlBAG2* take part in chloroplast breakdown and leaf senescence by modulating gene expression and generating reactive oxygen species (ROS) [23]. The overexpression of *OsBAG4* in rice induced autoimmunity and PCD, enhancing resistance to disease infection [24]. *GmBAG6-1* had higher transcript levels in SCN (soybean cyst nematode)-resistant soybean lines compared to susceptible ones [25]. The overexpression of *MusaBAG1* in bananas increases their resistance to the fungus *Fusarium oxysporum* f. sp. cubense (Foc) [26]. Early flowering was induced in transgenic overexpression lines of *HSG1* (grapevine BAG) in *Arabidopsis* by activation of the flowering promoter [27]. Taken together, earlier research suggested BAG genes are involved in a variety of functions. However, most of the current knowledge of BAG functions is centered on the model plant species.

It is crucial to investigate their roles in economically significant crops, as this knowledge could greatly enhance our agricultural practices and yield. This study analyzed the BAG gene family in passion fruit using diploid genome and transcriptome data, aiming to explore the role of BAG genes in stress response pathways. These findings will provide a robust basis for understanding regulatory variations essential for the genetic enhancement of passion fruit traits.

## 2. Methods

### 2.1. Identification of the Passion Fruit BAG Genes

The yellow passion fruit genome assembly (YPF) from the NGDC https://ngdc.cncb.ac.cn/gwh/Assembly/29473/show (accessed on 1 September 2024) [28] was used as a reference genome. The NGDC database was also used to download the genome data of the BXG and ZX genome assemblies [29,30] (https://ngdc.cncb.ac.cn/gwh/Genome/557/show (accessed on 1 September 2024)) to estimate the presence of BAG gene family members across different ecotypes. Previously reported 7 AtBAGs and 6 OsBAG protein sequences were used as combined input files to build BAG family signature Hidden Markov Model (HMM) files in the HMMER3.0 tool [31]. Later, this BAG profile HMM was used as a query to search against the whole protein database of yellow passion fruit to identify passion fruit BAG gene family members at a default threshold of E-value < 1 × 10^−5^. Eighteen putative PeBAG sequences above the threshold were identified in the HMMER tool. In comparison, further screening for the presence of the BAG domains in the NCBI Conserved Domain Database (https://www.ncbi.nlm.nih.gov/Structure/cdd/wrpsb.cgi (accessed on 10 September 2024)) and the HMMER web server (http://hmmer.org (accessed on 12 September 2024)) led to the exclusion of three protein sequences as false positives. Hence, 15 PeBAG protein sequences were confirmed as members of the gene family.

The physicochemical properties of PeBAG proteins were predicted using the EXPASY ProtParam tool (https://web.expasy.org/protparam/ (accessed on 14 September 2024)), while subcellular localization was estimated through WoLF PSORT (https://www.genscript.com/wolf-psort.html (accessed on 15 September 2024)).

### 2.2. Phylogenetic Analysis of the Passion Fruit BAG Genes

Phylogenetic analysis was performed in a Linux environment, ensuring high accuracy and fast computation compared to Graphical User Interface (GUI)- based software. The Mafft tool [32] was used for multiple sequence alignment of 7AtBAG, 6 OsBAGs, and 15 PeBAG protein sequences. ModelFinder [33] was used to find a fast model selection for accurate phylogenetic estimates. IQ-TREE [34] was employed to generate the maximum-likelihood phylogenetic tree, while UFBoot2 [35] improved the ultrafast bootstrap approximation. The accuracy was evident through the consistent distribution of proteins across all the phylogenetic trees generated.

### 2.3. Conserved Domains and Motif Analysis

The characteristic BAG domain features and conserved protein motif information were extracted from the NCBI CDD and MEME servers (https://meme-suite.org/meme/tools/meme (accessed on 10 September 2024)), respectively, employing PeBAG protein sequences as queries and depicted through TBtools software v2.326 [36].

### 2.4. Gene Locations, Duplications, and Synteny Analysis of PeBAGs

The distribution of PeBAGs on nine passion fruit chromosomes was ascertained from Genomic feature files and depicted using TBtools. Similarly, duplication and synteny analysis were performed using TBtool software.

### 2.5. Heatmap Clustering of Expression Data

Transcriptome data of *PeBAGs* were obtained from the Passionfruit Genomic database (http://passionfruit.com.cn/ (accessed on 20 September 2024)) [37], and heatmap clustering was performed in TBtools. The heatmap was based on log2 values and normalized by row. The similarity analysis of gene expression was based on Euclidean distances, and the complete linkage method was used for hierarchical clustering.

### 2.6. Promoter Analysis of PeBAGs and Prediction of Cis-Regulatory Elements

Whole genome gene promoter sequences of passion fruit were first extracted through the GTF/GFF3 sequences extract feature of TBtools. Later, the 2kb promoter sequences upstream of the transcription start sites of *PeBAGs* in fasta format were fed to the Multiple Em for Motif Elicitation (MEME) suite (http://meme-suite.org/tools/meme (accessed on 25 September 2024)) to identify novel motifs. Six minimum motifs were identified using zoops motif site distribution. The conserved motifs were later analyzed through TOMTOM (https://meme-suite.org/meme/tools/tomtom (accessed on 1 October 2024)) against already known motifs in JASPAR CORE (2024) plants and *Arabidopsis thaliana* for plant gene promoters. Finally, the patterns of transcription factor binding elements on the promoters were illustrated.

### 2.7. Heat Stress Treatments, RNA Extraction, and qRT-PCR

The cultivation of passion fruit seedlings, stress treatments, RNA extraction, and qRT-PCR assays were performed as methods described in Shad et al. 2024 [38]. Yellow passion fruit local cultivar stem cuttings were sown in pods containing a compost-vermiculite mix. Two-month-old seedlings with healthy growth were put in the growth chamber. For heat stress, the temperature was set at 40 °C. Heat-treated leaf samples were collected after 0, 6, 12, and 24 h intervals. For each interval, nine seedlings were used in total, and three replicates were used as each replicate contained three seedlings (biological replicates). After stress treatments, the leaves were frozen, and the RNAs of all samples were extracted employing a Vazyme RNA isolation kit, Vazyme Biotech Co., Ltd., Nanjing, China. cDNA was synthesized from the extracted RNA using Aidlab Truescript 1st Strand cDNA Synthesis Kit (Aidlab Biotechnologies, Ltd., Beijing, China). The DLAB accurate96 system was employed for Real-time PCR using the PC59-2 × SYBR Green qPCR Mix (Aidlab Biotechnologies, Ltd., Beijing, China), with three technical replicates, and primers are listed in Table S4. The qRT-PCR conditions were 95 °C for 2 min, 40 cycles of 95 °C for 15 s, 60 °C for 30 s, and 72 °C for 30 s. Three technical replicates from three biological replicates were used for each analysis, and the 2^–∆∆Ct^ method was used to determine the fold change in each gene. For normalization of mRNA levels, the passion fruit *PeEF1a* gene was used as an internal control or reference [39].

### 2.8. GFP Subcellular Localization

The GFP vector construction, agroinfiltration, and confocal microscopy for GFP signals of three PeBAG proteins were performed according to the methods described in Huang et al. (2024) [40]. The single-fragment cloning primers were designed employing the Vazyme tool (https://crm.vazyme.com/cetool/en-us/singlefragment.html (accessed on 4 October 2024)). pCAMBIA1300-35S-GFP vector, which possesses a 35S constitutive promoter, linearized with a single restriction enzyme (*BamH*I) was used for transient expression. The coding sequences of *PeBAG2-1*, *2-2*, and *4-1* without stop codon were amplified from the cDNA using primers listed in Table S5 through high-fidelity P525 polymerase. The amplified fragments were cloned into linearized pCAMBIA1300-35S-GFP using the Uniclone One Step Seamless Cloning Kit (SC612). The cloned vectors were introduced into competent cells of the DH5α strain of *E. coli* through the heat-shock method. Following the identification of positive colonies through colony PCR, sequencing was performed to confirm the presence and orientation of the CDS. The plasmid extraction was performed using an Invitrogen Plasmid Extraction Kit. The empty and cloned vectors were introduced into *Agrobacterium tumefaciens* strain GV3101 and co-transformed with chromatin marker (H2B:mCherry) and chloroplast marker into *Nicotiana benthamiana* leaves. The transformed plants were kept in darkness for 60 h periods. The leaves were taken after 60 h, and using a confocal laser scanning microscope (FV12000MPE, Olympus, Tokyo, Japan), the fluorescence signal was observed as mCherry and chloroplast marker emitted light at 600–650 nm and was excited at 552 nm, while GFP emitted light at 500–530 nm and was excited at 488 nm.

## 3. Results

### 3.1. Identification of the Passion Fruit BAG Gene Family

In this study, 15, 12, 9, and 7 *PeBAGs* were identified in the YPF (Table S1), Passion Fruit Genomic Networks database, and BXG and ZX genome assemblies, respectively (Table S2). Additionally, we estimated the physiochemical properties of PeBAGs (Table 1). The protein encoded by *PeBAGS* ranged from 157 (PeBAG6) to 518 (PeBAG7) amino acids. The molecular weight (MW) of the encoded proteins ranged from 1.79 kDa (PeBAG6) to 5.67 kDa (PeBAG7). The isoelectric point (pI) varied from 4.69 (PeBAG7) to 9.82 (PeBAG8). PeBAG5/6/11/12 were estimated to be stable in a test tube, while the rest were estimated to be unstable. All the proteins exhibited negative values for the grand average of hydropathicity (GRAVY) parameters. Additionally, subcellular localization prediction estimated that most PeBAGs are localized to the nucleus, cytoplasm, and chloroplasts.

### 3.2. Phylogenetic Classification of PeBAGs Genes Encoded Proteins

**Figure 1:**
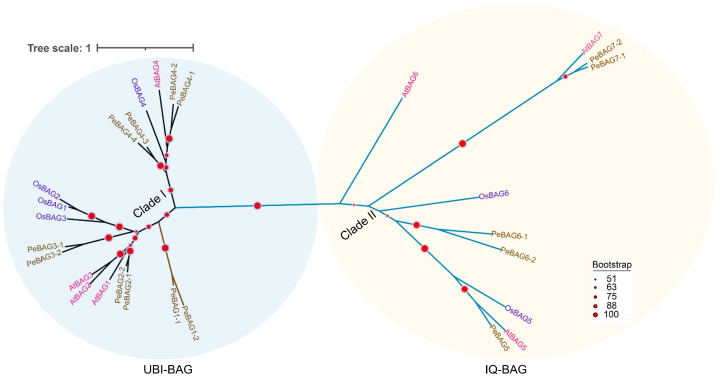
Phylogenetic analysis of BAG proteins from passion fruit, rice, and Arabidopsis. The bootstrap replicate values are proportional to the radius of the red circle, as depicted in the bottom right part of the figure.

The fifteen identified PeBAGs, seven AtBAGs, and six OsBAGs were used to build a phylogenetic tree to examine the evolutionary interrelationships and classification of BAG proteins in passion fruit (Figure 1). Based on the phylogenetic relationships, PeBAGS were classified into two major clades: clade I (PeBAG1-1/2, 2-1/2, 3-1/2, and 4-1/2/3/4) and clade II (PeBAG5, 6-1/2, and 7-1/2). The orthologous proteins of Arabidopsis and rice in each clade were also inferred. Arabidopsis AtBAG1/2/3/4 and rice OsBAG1/2/3/4 were clustered into Clade I, while AtBAG5/6/7/8 and OsBAG5/6 were distributed into Clade II, along with their passion fruit orthologs.

**Figure 2:**
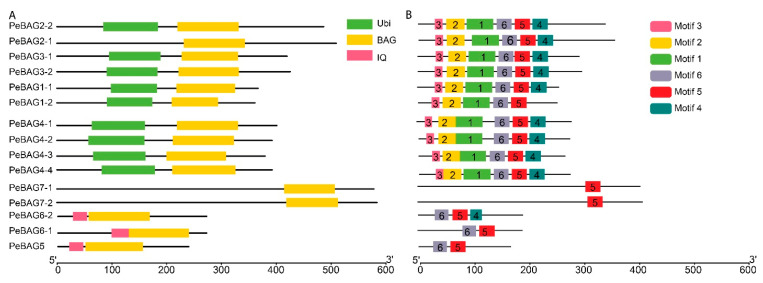
Conserved domains (**A**) and conserved motif (**B**) analysis of PeBAG proteins.

The domain analysis of PeBAG proteins identified three groups of proteins based on the presence of domains (Figure 2). Three members (PeBAG6-1/6-2/5) possessed the CaM-binding IQ domain and characteristic BAG domains, while two members (PeBAG7-1/7-2) of this gene family only had BAG domains (Figure 2A). The rest of the PeBAGs possessed Ubiquitin domains in addition to the canonical BAG domains.

Generally, BAG proteins are diversified into two groups based on their structural properties and conserved domain features [41]. The first group features a ubiquitin-like (UBL) domain at the N-terminus, similar to mammalian BAG1. In contrast, a plant-specific CaM-binding IQ motif near the BAG domain characterizes the second group. AtBAG1/2/3/4 are homologous to animal BAG1 and belong to group 1. Clade II members of Figure 1 belonged to the IQ Calmudlin binding group of BAG proteins, while Clade I members belonged to the Ubiquitin domain-containing BAG protein group. Motif analysis identified six motifs across PeBAG proteins (Figure 2B). The Ubi-BAG domain group proteins possessed all six motifs, while the IQ-BAG group contained motifs 4, 5, and 6. The only BAG domain-containing protein had motif 5, suggesting this motif was conserved across all the gene family members.

### 3.3. Chromosomal Locations, Duplications, and Synteny Analysis of PeBAGs

**Figure 3:**
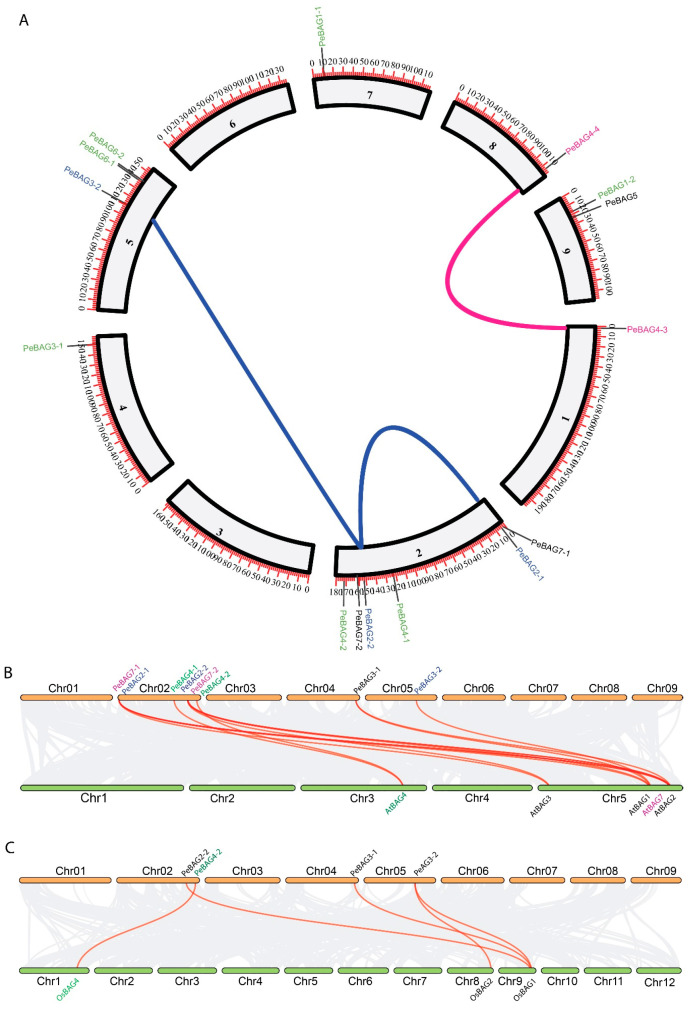
Chromosomal locations and duplication of PeBAGS within the passion fruit genome (**A**). Syntenic relationships of PeBAGs between (**B**) Passion fruit and Arabidopsis, and (**C**) Passion fruit and rice. The paralog gene pairs in (**A**) have been labeled and connected with lines in the same color. Horizontal orange bars indicate passion fruit chromosomes in (**B**,**C**), while red connecting lines depict the collinear gene pairs.

As shown in Figure 3A, PeBAGs exhibited an uneven distribution across the nine linkage groups. Of these chromosomes, 2 possessed the highest number of 6 genes, followed by chromosomes 5 and 9, which contained 3 and 2 *PeBAGs*, respectively. Chromosomes 1, 4, 7, and 8 each accommodated one gene. Linkage groups 3 and 6 did not possess any *PeBAG*.

Gene duplication is an essential factor in the expansion of genes and the emergence of new gene functions. In this study, duplication analysis was performed through the MCScan plugin in TBtools. The analysis identified three pairs of duplicated genes among fifteen genes (Figure 3A). Interestingly, *PeBAG2-2* was common between two separate duplicated pairs with *PeBAG2-1* and *PeBAG3-2*, suggesting it may be a progenitor gene. The third pair of paralogs included *PeBAG4-3* and *PeBAG4-4*. Furthermore, all duplication events were either categorized as whole-genome duplications (WGDs) or segmental, and no instances of tandem duplications were observed. The analysis summarized that seven genes originated from whole-genome duplication (WGD) or segmental duplication, while eight genes were singletons (Table S3).

Synteny analysis was performed between passion fruit, Arabidopsis (a dicot), and rice (a monocot) to identify collinear gene pairs (Figure 3B,C). The study identified 13 and 5 collinear gene pairs of passion fruit with Arabidopsis and rice, respectively. *PeBAG2-1*/*2-2*/*3-1*/*3-2*, *4-1*/*4-2*, and *7-1*/*7-2* corresponded to the *AtBAG1*/*2*/*3*, *4*, and 7, respectively (Figure 3B). Similarly, *PeBAG3*-*2*, *3-1*, *2-2,* and *4-2* formed orthologous pairs with rice *OsBAG1/2*, *1*, *1*, and *4,* respectively (Figure 3C). Several *PeBAG* genes exhibited multiple collinear pairs, where one gene from passion fruit corresponded to several genes in Arabidopsis or rice, or vice versa. For example, *PeBAG2-2* was linked with *AtBAG1/2/3* and *OsBAG1*, suggesting that these genes may play a crucial role in the evolution of the BAG gene family, and may be present before the speciation events that led to the distinct lineages of Arabidopsis and rice.

**Figure 4:**
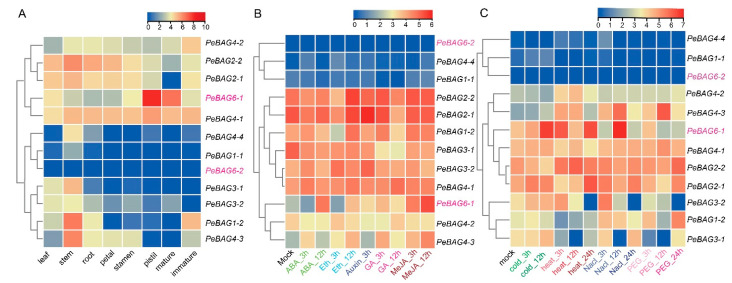
Expression profiles of PeBAGs based on FPKM data. (**A**) Across different tissues of passion fruit, (**B**) after various hormonal applications, and (**C**) after exposure to cold, heat, salt, and drought stress. The scale on the top right of each figure panel depicts log2 fold changes in gene transcripts. The IQ-BAG domain clade II *PeBAGS* are displayed in pink, while the rest are denoted in black.

Transcriptome analysis of passion fruit *BAGs* was performed for different plant tissues, hormones, and abiotic stresses (Figure 4). The expression analysis revealed varied transcript accumulation patterns in the tissue for *PeBAGs* (Figure 4A). IQ-BAG type *PeTCP6-1* exhibited very high expression in the pistil and mature fruit. *PeBAG4-3/3-1/1-2* were upregulated in stems, while *PeBAG6-2/1-1/4-4* exhibited extremely low expression across all tissues. Interestingly, paralog gene pair *PeBAG2-1*/*2-4* was preferentially upregulated in vegetative tissues compared to the reproductive tissues. On the other hand, *PeBAG4-1* exhibited constitutive expression patterns. These results showed that *PeBAGs* might perform tissue-specific functions. *PeBAG6-1* exhibited highly induced expression in response to hormonal treatments with ABA and MeJA (Figure 4B). Similarly, *PeBAG4-3* exhibited upregulation in response to all hormonal treatments. *PeBAG6-1* was also strongly induced in response to cold, heat, and salt stress, while *PeBAG4-3* exhibited upregulation in response to drought and salt stress treatments (Figure 4C). *PeBAG2-1/2-2* also showed higher transcript accumulation in response to heat stress. *PeBAG12* was highly expressed after drought stress. These analyses suggested *PeBAG6-1/4-3/2-1/2-2/1-2* might be novel candidate genes for further genetic studies.

**Figure 5:**
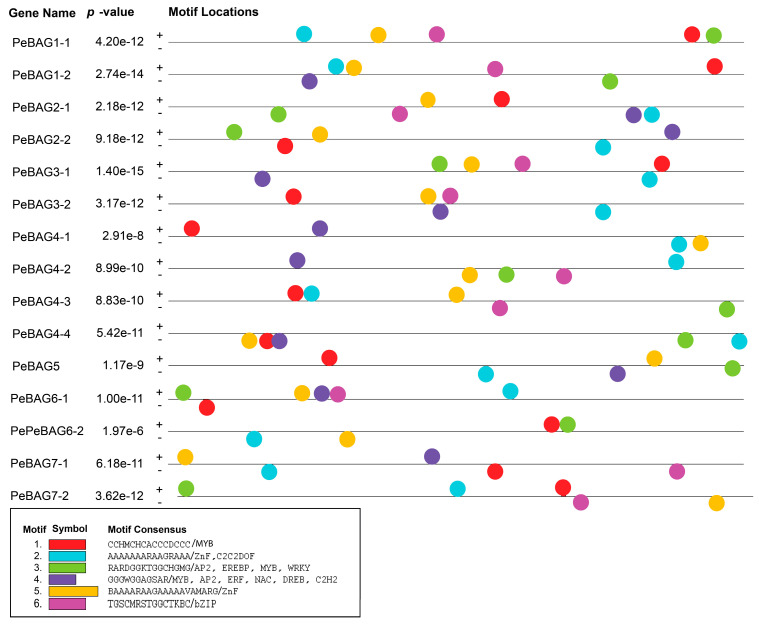
Transcription factor binding sites in *PeBAGs* promoters. The BAG gene family executes various functions by interacting with heat shock proteins (HSPs), acting as co-chaperones, or regulating chaperones during the response to heat stress and development [42,43]. To determine the importance of these genes, we examined the differential regulation of *PeBAGs* in passion fruit seedlings subjected to heat stress. The qRT-PCR assays revealed that *PeBAG1-1*/*1-2*/*3-1*/*3-2*/*4-3*/*4-4*/*6-1*/*6-2* were significantly upregulated in response to varying levels of heat stress, while *PeBAG4-1*/*4-2* exhibited downregulation, indicating a possible negative regulatory function during heat stress (Figure 6). Interestingly, paralogs *PeBAG2-1/2-2* did not show significant responses to heat stress treatments, suggesting that they may not be involved in high-temperature responses. Together, these results provide novel insights into the role of *PeBAG* in passion fruit’s heat stress response, providing a framework for further functional elucidation through transgenic validation, mutational analysis, and protein interaction assays.

Promoter analysis of *PeBAGs* identified conserved motifs that are involved in the binding of transcription factors. The analysis reported that almost all the PeBAGs promoters were enriched with MYB, ZnF, C2C2-DOF, Ap2, DREB, ERF, NAC, WRKY, and bZIP transcription factors (Figure 5).

**Figure 6:**
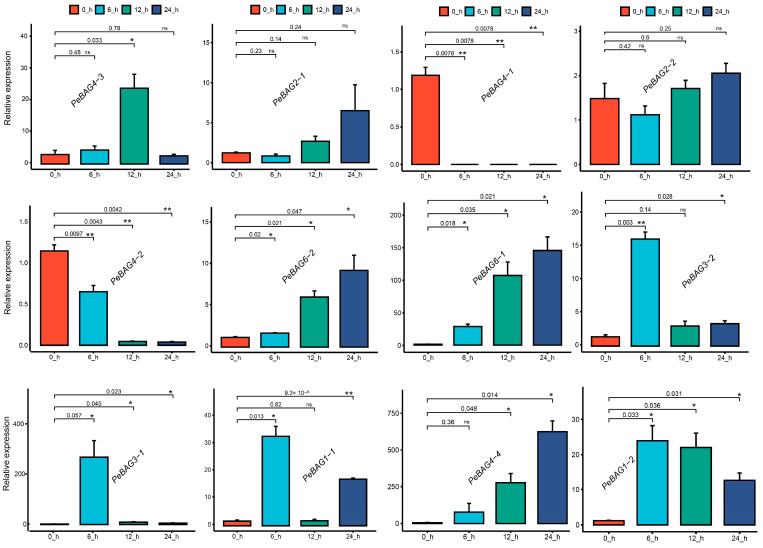
qRT-PCR validation of *PeBAGs* gene expression under heat stress. The X-axis depicts treatment levels, while the Y-axis represents the relative expression. Each bar represents the mean relative expression ± standard error (SE) from three independent biological replicates. Statistical analysis was performed using Student’s *t*-test, with significance levels indicated by asterisks (ns = non-significant, * *p* < 0.05, ** *p* < 0.01).

**Figure 7:**
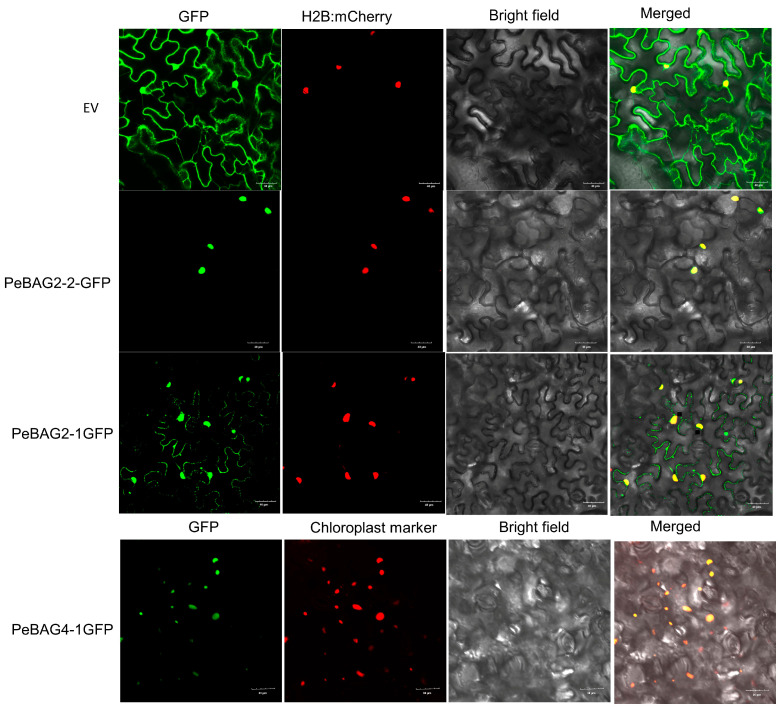
Subcellular localization of GFP-tagged PeBAGs in *Nicotiana benthamiana* leaves. The leaves were agroinfiltrated with *Agrobacterium* harboring the constructed PeBAG-GFP and empty GFP plasmid. Scale bar = 30 μm.

Subcellular localizations provide a crucial understanding of protein functions. For protein subcellular localization assays, three proteins, PeBAG2-1/2-2/4-1, were chosen because their Arabidopsis orthologs exhibited varied subcellular localizations (Figure 7). The plasmid containing these proteins, encoding CDS fused with a C-terminal GFP tag sequence, was agro-infiltrated into *Nicotiana benthamiana* leaves. The fluorescence assays revealed that PeBAG2-1GFP exhibited fluorescence in both the nucleus (coinciding with H2B:mCherry) and the cytoplasm (outlining the cell structure), indicating dual nuclear and cytoplasmic localization. PeBAG2-2GFP signals strongly overlapped with the nuclear marker, suggesting nuclear localizations. On the other hand, PeBAG4-1GFP exhibited strong colocalization with the chloroplast marker, confirming its localization in chloroplasts. PeBAG4-1 subcellular localization was in contradiction to the predicted location in the nucleus (Table 1); however, we reason that the prediction tool suggested localization based on scores at multiple locations, such as 4 for the nucleus and 2 for the chloroplast, while we only listed the highest scoring location in Table 1.

**Figure 8:**
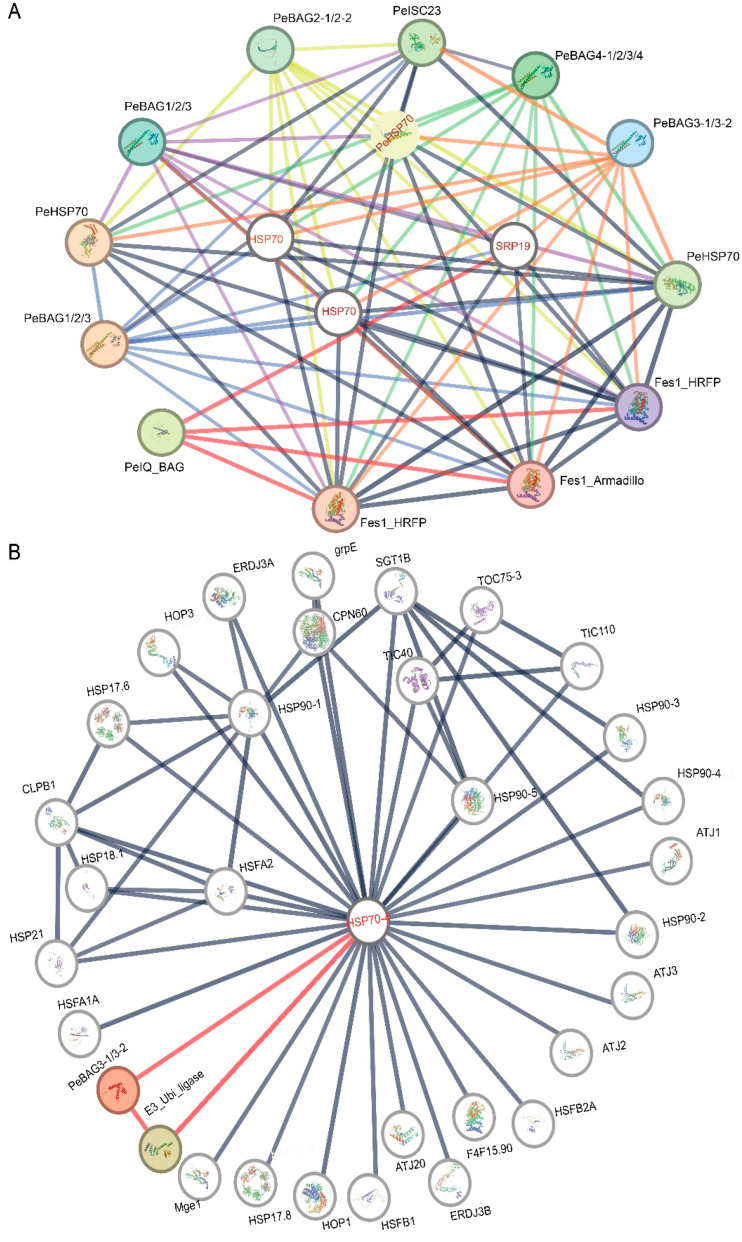
Protein–protein interaction network of PeBAGs based on (**A**) rice and (**B**) *Arabidopsis* orthologs.

Using the rice and Arabidopsis orthologs of PeBAGs, protein–protein interaction networks were predicted at STRING version 12 at a high confidence level (>0.7) based on co-expression and experimental interactions (Figure 8). The predicted interaction proteins of PeUBI-BAGs, based on rice orthologs (Figure 8A), mainly included HSP70s, the Armadillo/beta-catenin-like repeat family (with Ubiquitin ligase function), SRP19 (Signal recognition particle 19 kDa protein), and heat repeat family proteins (Fes1 domain). Contrarily, IQ-BAG lacked the predicted interaction with HSP70s; however, their interaction with the Fes1 domain and SRP19 was identified. Similarly, based on Arabidopsis orthologs (Figure 8B), UBI-BAG (PeBAG3) directly interacted with E3 Ubiquitin ligase and HSP70 proteins.

## 4. Discussions

Passion fruit is a rich source of vitamins, carbohydrates, amino acids, minerals, and essential elements [44]. Moreover, it is enriched with bioactive substances, including phenolic acids, flavonoids, and antioxidants, which are used in traditional Chinese medicine due to their medicinal benefits [45]. Although passion fruit is native to tropical climates, heat stress can lead to stunted growth, fewer floral buds, a lower fruit set rate, a shorter flowering period, increased rates of fruit drop, and malformed fruit [46]. The repertoire of the BAG gene family, which plays pivotal roles in conferring resistance against abiotic and biotic stresses, has been identified in various plant species, including Arabidopsis [47], rice [48], tomato [20], maize [17], sugarcane [49], pigeonpea [43] and banana [50]. Identification of heat-stress-responsive candidate genes for functional studies can aid in selecting heat-resistant genotypes. This study provides a comprehensive identification, characterization, and expression analysis of *PeBAGs*. Passion fruit BAG gene family members, along with previously reported Arabidopsis and rice orthologs, were phylogenetically clustered into two clades (Figure 1) in agreement with the previous studies [47,48]. Notably, passion fruit possessed more BAG genes (15) than seven and eight genes in rice and Arabidopsis, respectively, indicating an expansion of the gene family through duplications.

The characteristic BAG domain, located near the C-terminus of BAG proteins, interacts with the ATPase domain of the HSP70 protein, which is essential for its co-chaperone activity [10]. Similarly, all the PeBAGs studied in our research possessed the canonical BAG domain. The domain organizations revealed that, in addition to the characteristic C-terminal BAG domains, nine PeBAGs possess N-terminal ubiquitin domains, and three contain plant-specific calmodulin-binding domains. E1, E2, and E3 ligases ubiquitinate the ubiquitin-like domain found in BAG proteins, which ultimately leads to degradation by autophagy or proteasomal degradation [24]. The combined evolutionary analysis, encompassing phylogenetics, conserved domains and motifs, duplication, and collinearity, revealed the presence of four distinct orthologous groups among the studied species. Group 1 (UBI-BAG) comprises At/Os/PeBAG1, At/Os/PeBAG2, and At/Os/PeBAG3; Group 2 consists of At/Os/PeBAG4 orthologs; Group 3 (IQ-BAG) includes At/Os/PeBAG5 and At/Os/PeBAG6, and the fourth group contains At/PeBAG7 orthologs. The IQ motif can bind Calmodulin (CaM) and influence the formation of a complex between CaM and the target protein [51]. AtBAG5 (IQ-BAG) has been linked to the control of senescence of leaves reliant on reactive oxygen species (ROS), probably through calcium signals. AtBAG5’s location in the mitochondria and calmodulin’s ability to bind calcium may indirectly maintain the electric potential of the mitochondrial membrane [51,52,53]. CaM/AtBAG5/Hsc70 forms a tripartite signaling network that regulates the shielding effect of Ca^2+^ on leaf senescence by maintaining high mitochondrial Ca^2+^ levels, which inhibit senescence [51].

Gene duplication processes are crucial for the expansion of gene families, significantly enhancing their functional diversity and evolutionary adaptability [54,55]. This study identified three paralogous *PeBAG* gene pairs distributed across four passion fruit chromosomes. All these duplication events were segmental or whole-genome duplication (WGD), which may relate to a comparatively recent WGD event in passion fruit, estimated to have occurred around 40 million years ago [28]. Compared to Arabidopsis and rice, the passion fruit BAG genes underwent significant expansion in PeBAG1/2/3/4/6/7, while recent research in maize reported similar results in *ZmBAG1*/*4*/*7* orthologs [17]. This extension may result from selection pressures on passion fruit, thereby improving its capacity to flourish and adapt to various environmental extremes in a range of habitats [55]. In addition, the conservation of *BAG* genes between passion fruit, rice, and *Arabidopsis* was revealed by synteny analysis (Figure 3B,C). As a sign of evolutionary conservation, this suggests that these genes have been inherited from shared ancestors and have preserved identical genomic loci [56,57].

Our research revealed that all *PeBAG* genes exhibit distinct patterns of a variety of transcription factor binding (Figure 5). The promoters of *PeBAGs* were highly enriched with MYB-associated motifs, aligning well with previous findings [50]. Similarly, the presence of bZIP and WRKY transcription factor binding sites in PeBAG promoters might indicate a parallel regulatory module as discovered in Arabidopsis, where AtBAG7 interacts with AtBiP2 (a paralog of HSP70) and ER-membrane AtbZIP28 in response to ER stress [58]. Heat stress mediates the release of AtBAG7 from the complex and translocation to the nucleus, where it interacts with WRKY29. Stress tolerance mechanisms are activated when AtBAG7 promoters are bound by WRKY29, which triggers the transcription of AtBAG7 and other chaperons [59]. While BAG genes are known to possess multiple TF binding motifs and are stress-responsive, direct experimental evidence for specific upstream transcription factor regulators remains scarce, posing an opportunity for further studies.

The differential expression patterns of *PeBAGs* in response to abiotic stressors and hormonal applications further highlight the potential involvement of *PeBAGs* in plant responses to stress (Figure 4). The transcriptome analysis revealed the specific upregulation of *PeBAG6-1* in pistils and mature fruits, *PeBAG1-2/3-1/4-3* in the stem, and *PeBAG2-2* in vegetative organs, suggesting their particular roles in passion fruit organ growth and development. *PeBAG6-1* exhibited highly induced expressions for hormonal responses following ABA and MeJA treatments, while *PeBAG4-3* exhibited preferential upregulation for all hormone applications. Abiotic stresses such as cold, heat, salt, and drought enhanced the transcripts of the *PeBAG3-1*/*3-2*/*6-1*, *PeBAG2-1*/*6-1*, *PeBAG3-2*/*4-3*/*6-1,* and *PeBAG1-2/4-3*, respectively. In plants, BAG4 targets the KAT1 potassium channels and regulates the stomatal movements, which has a profound impact on water use efficiency and drought tolerance [60]. We performed qRT-PCR assays of PeBAG genes after heat stress treatments to verify transcriptome data results (Figure 6). *PeBAG1-1/1-2*, *PeBAG3-1*/*3-2*, *PeBAG4-3*/*4-4*, and *PeBAG6-1*/*6-2* were significantly upregulated. *PeBAG4-1* and *PeBAG4-2* showed downregulation, consistent with the in silico gene expression data for Arabidopsis *AtBAG4*, which is also downregulated under heat stress [18]. *PeBAG6-1* exhibits high differential expression across a range of abiotic stresses and hormonal treatments, paralleling the role of its Arabidopsis ortholog AtBAG6, which has been reported to be involved in ABA responses and heat stress regulation in multiple studies [16].

Understanding the biological functions of proteins is aided by studying their subcellular locations. In this research, PeBAG proteins were found to exhibit subcellular localization signals at various organelles (Figure 7). For example, PeBAG2-1, 2-2, and 4-1 showed GFP signals in the nucleus and cytoplasm, as well as the nucleus and plastids, respectively, indicating their functional divergence. Varied subcellular localizations of BAG proteins have been reported in *Arabidopsis*, tomato, rice, wheat, and soybean species as well. Earlier studies reported that OsBAG4 [21], AtBAG4 [60], SlBAG2/9/5b [23,61], and TaBAG/TaBAG2 [42] were localized to both the cytoplasm and the nucleus. Further studies are required to help establish links between candidate *PeBAG* genes identified in this research and their phenotypic or stress responses. Our results provide insights into functional analysis of *PeBAG* genes, and a few of them (*PeBAG4-3* and *6-1*) would be promising targets for the genetic improvement of heat stress tolerance of passion fruits.

## Figures and Tables

**Table 1:** Basic information on the passion fruit BAG gene family members.

Gene Name	Locus ID	Chromosome	Size (aa)	MW (Da)	pI	A.I.	Stability	GRAVY	Predicted Location
PeBAG1-1	Pe6g01112	LG6	257	29,041.22	8.92	88.33	Unstable	−0.445	Nucleus
PeBAG1-2	Pe9g01431	LG9	251	28,073.24	6.37	88.45	stable	−0.324	chloroplast
PeBAG2-1	Pe2g00028	LG2	348	38,366.55	9.59	75.89	Unstable	−0.643	Nucleus
PeBAG2-2	Pe2g02075	LG2	343	37,833.91	9.57	72.16	Unstable	−0.684	Nucleus
PeBAG3-1	Pe4g00348	LG4	294	33,283.48	9.42	87.79	Unstable	−0.652	Cytoplasm
PeBAG3-2	Pe3g01971	LG3	298	34,012.46	9.82	90.23	Unstable	−0.584	Cytoplasm
PeBAG4-1	Pe2g01777	LG2	281	31,512.85	4.82	73.06	Unstable	−0.92	Nucleus
PeBAG4-2	Pe2g03293	LG2	275	30,762.77	5.64	74.73	Stable	−0.675	Cytoplasm
PeBAG4-3	Pe1g00342	LG1	295	33,205.96	5.88	79.59	Unstable	−0.499	Nucleus
PeBAG4-4	Pe7g01275	LG7	275	30,808.53	8.79	91.09	Stable	−0.417	chloroplast
PeBAG5		LG9	167	18,975.48	5.49	81.74	unstable	−0.535	chloroplast
PeBAG6-1	Pe3g01508	LG3	518	56,789.93	4.69	74.46	Unstable	−0.726	Nucleus
PeBAG6-2	Pe3g01506	LG3	157	17,927.22	5.21	83.89	Stable	−0.89	chloroplast
PeBAG7-1		LG2	409	47,230.17	8.95	83.47	unstable	−0.634	chloroplast
PeBAG7-2		LG2	405	46,606.38	9.47	78.57	unstable	−0.65	Cytoplasm

## Data Availability

The original contributions presented in this study are included in the article/Supplementary Material. Further inquiries can be directed to the corresponding authors.

## Supplementary Materials

The following supporting information can be downloaded at: https://www.mdpi.com/article/10.3390/plants14182887/s1, Table S1. Identification of passion fruit BAG gene family members through HMMER 3.0. Table S2. Locus IDs of BAG genes on different genome assemblies of passion fruit. Table S3. Evolutionary origin of the *PeBAGs* determined through gene duplication analysis. Table S4. List of primers used for qRT-PCR. Table S5. Primers used for GFP subcellular localizations using BamHI for restriction cloning.
