# Supplementary material for: Evolution and Functional Dynamics of the BAG Gene Family in Passion Fruit (Passiflora edulis)
Source: Plants (Basel). 2025 Sep 17;14(18):2887. doi: 10.3390/plants14182887 (PMC12473569; doi:10.3390/plants14182887)
Supplement: Supplementary file 1 [file plants-14-02887-s001.zip › plants-3754555-supplementary.pdf]

Table S1. Identification of passion fruit BAG gene family members through HMMER 3.0.

Sequence	E-value	score
Pes02T0242400SH	4.2E-72	244.6
Pes02T0026000SH	3.5E-63	215.1
Pes02T0019900SH	9.1E-49	167.4
Pes02T0248800SH	3.2E-48	165.6
Pes01T0033100SH	7.6E-46	157.7
Pes04T0271900SH	3.1E-43	149.1
Pes05T0125600SH	5.2E-42	145
Pes07T0072200SH	5.9E-42	144.9
Pes08T0148100SH	1.3E-40	140.4
Pes02T0201600SH	1.5E-40	140.2
Pes02T0377100SH	5.2E-37	128.5
Pes09T0148100SH	2.3E-31	109.8
Pes05T0180500SH	2.9E-27	96.2
Pes05T0180600SH	4.2E-25	89.1
Pes09T0237900SH	4.7E-25	88.9
Pes06T0361400SH	3E-12	46.5
Pes06T0384100SH	1.2E-09	38
Pes03T0030000SH	5.9E-09	35.6

Table S2. Locus IDs of BAG genes on different genome assemblies of passion fruit.

Gene names	PPF	PSGN	BXG	ZX
PeBAG1-1	Pes07T0072200SH	Pe6g01112	GWHPAZTM015593	no hit
PeBAG1-2	Pes09T0148100SH	Pe9g01431	GWHPAZTM019129	no hit
PeBAG2-1	Pes02T0026000SH	Pe2g00028	GWHPAZTM002915	GWHPANWG006425
PeBAG2-2	Pes02T0242400SH	Pe2g02075	GWHPAZTM002915	GWHPANWG006425
PeBAG3-1	Pes04T0271900SH	Pe4g00348	no hit	no hit
PeBAG3-2	Pes05T0125600SH	Pe3g01971	GWHPAZTM010346	GWHPANWG026396
PeBAG4-1	Pes02T0201600SH	Pe2g01777	GWHPAZTM001587	GWHPANWG006983
PeBAG4-2	Pes02T0377100SH	Pe2g03293	GWHPAZTM001587	GWHPANWG006674
PeBAG4-3	Pes01T0033100SH	Pe1g00342	GWHPAZTM007476	GWHPANWG013991
PeBAG4-4	Pes08T0148100SH	Pe7g01275	GWHPAZTM017767	GWHPANWG013991
PeBAG5	Pes09T0237900SH		no hit	no hit
PeBAG6-1	Pes05T0180500SH	Pe3g01508	GWHPAZTM009996	GWHPANWG032482
PeBAG6-2	Pes05T0180600SH	Pe3g01506	no hit	no hit
PeBAG7-1	Pes02T0019900SH		GWHTAZTM001180	GWHPANWG009287
PeBAG7-2	Pes02T0248800SH		GWHTAZTM001180	GWHTANWG009287

Table S3. Evolutionary origin of the *PeBAGs* determined through gene duplication analysis.

Gene name	Origion	Gene name	Origion
PeBAG1-1	Singleton	*PeBAG2-1*	WGD or Segmental
PeBAG1-2	Singleton	*PeBAG2-2*	WGD or Segmental
PeBAG3-1	Singleton	*PeBAG7-1*	WGD or Segmental
PeBAG4-1	Singleton	*PeBAG7-2*	WGD or Segmental
PeBAG4-2	Singleton	*PeBAG4-3*	WGD or Segmental
PeBAG5	Singleton	*PeBAG4-4*	WGD or Segmental
PeBAG6-1	Singleton	*PeBAG3-2*	WGD or Segmental
PeBAG6-2	Singleton		

Table S4. List of primers used for qRT-PCR.

Gene Name	Primer sequence
qBag1-1F	ACAGTCAATTCTCAGGCCAG
qBag1-1R	AAGTATTCCCCATTTTCCCTCTC
qBag1-2F	GAGGTACATGGAGATGCGTAAG
qBag1-2R	TCTGAACTTCTGCCACCTTG
qBag2-1F	GGTGTCGGCTCTTGAATCTATC
qBag2-1R	CACAATTCCGTCTAACTTCAGC
qBag2-2F	TGTCGGCTCTTGAATCTGTG
qBag2-2R	ATCACCCATAATTCCGTCCAG
qBag3-1F	CGTTTATGCCGACAAATAAGAGG
qBag3-1R	TTCTGAACTAACATCCCACCTG
qBag3-2F	CGGTGAAGGCAAAGATTAAAGG

qBag3-2R	TCCTCTGTACTAACATCCCTCC
qBag4-1F	CATGTCCCTGCTCAATCTACC
qBag4-1R	TTATCTTTCACACCTGCCTCG
qBag4-2F	ATTCGAGCACCCGTTATGAG
qBag4-2R	CTTCGTCCCTTACCTGTACAAG
qBag4-3F	TCCTGCTCAATCCACTTTCG
qBag4-3R	GCCTCTGAATAGCAACCTCTG
qBag4-4F	GGGATTGAGGTTGGTCGTATC
qBag4-4R	ACTTAGGCTTCCAACACCAG
qBag6-1F	AGGAAAGAGAAACCGTTGGAG
qBag6-1R	TCGCCTTCATCTCCTTCAATC
qBag6-2F	AATGACAAGATCGGAAGGGC
qBag6-2R	AACCTAATCCACACCCTTTCG

Table S5. Primers used for GFP subcellular localizations using BamHI for restriction cloning.

**Primer name**	**Sequences**
PeBAG2-1F	gagctcggtacccggggatccATGATGAGAACGAAGACTAAACCGA
PeBAG2-1R	catgtcgactctagaggatccATCGTAGAAATCCCAAGGGAATT
PeBAG2-2F	gagctcggtacccggggatccATGATGAGAATGAAAACTAAACCTGC
PeBAG2-2R	catgtcgactctagaggatccATCGAAGAAATCCCACGGGA
PeBAG4-1F	gagctcggtacccggggatccATGAAGAATTCCAGTTCTAGTAATGAAGA
PeBAG4-1R	catgtcgactctagaggatccATCAAACCTTTCCCAGTCCTCA
